# Fraturas por compressão vertebral: fatores preditivos de falha na cifoplastia de aumento vertebral

**DOI:** 10.1055/s-0046-1817023

**Published:** 2026-03-25

**Authors:** Joana Araújo de Azevedo, Vasco Campos, Paulo Gil Ribeiro, Nuno Oliveira, Pedro Varanda, Bruno Direito-Santos

**Affiliations:** 1 Departamento de Cirurgia Ortopédica, Hospital de Braga, Braga, Portugal; 2 Escola de Medicina, Universidade do Minho, Braga, Portugal

**Keywords:** cifoplastia, cimento ósseo, coluna vertebral, fraturas por compressão, bone cements, fractures, compression, kyphoplasty, spine

## Abstract

**Objetivo:**

Identificar fatores preditivos de falha da cifoplastia em pacientes com fraturas por compressão vertebral (FCV), o que pode auxiliar na tomada de decisões clínicas e otimizar os resultados terapêuticos.

**Métodos:**

Este estudo de coorte retrospectiva incluiu pacientes com FCV submetidos à cifoplastia e categorizados com base na presença ou ausência de falha do tratamento. Foram avaliados 40 prontuários com tempo mínimo de acompanhamento de 6 meses; 6 casos foram excluídos por não atenderem aos critérios de inclusão. As variáveis analisadas foram idade, sexo, tempo até a cirurgia, nível da fratura, pontuação do Sistema de Classificação de Lesões da Coluna Vertebral da AO e escore de fraturas osteoporóticas (OF). Parâmetros radiológicos, como altura do corpo vertebral e ângulos cifóticos regionais e segmentares, foram medidos antes e após a cirurgia. Além disso, a proporção do corpo vertebral ocupada por cimento foi analisada, com ênfase em sua distribuição anterior e posterior.

**Resultados:**

Dentre os 34 pacientes, 20 (58,8%) apresentaram resultados satisfatórios, ou seja, ausência de critérios de falha até o último acompanhamento (grupo 1), enquanto 14 (41,2%) apresentaram falha (grupo 2). A falha foi definida pela presença de fratura vertebral adjacente, colapso vertebral recorrente ou recidiva de dor incapacitante. A cifoplastia gerou melhora significativa na altura do corpo vertebral e nos ângulos cifóticos. À análise de regressão, apenas a porcentagem de distribuição posterior do cimento emergiu como fator preditivo independente de falha (razão de chances ajustada = 1,684; *p* = 0,012). O aumento do ganho de altura vertebral posterior indicou tendência à falha, enquanto o ganho de altura anterior pareceu ter efeito protetor.

**Conclusão:**

A maior porcentagem de cimento localizada posteriormente no corpo vertebral está associada a um aumento do risco de falha da cifoplastia. Esses achados destacam a importância dos padrões de distribuição do cimento no planejamento cirúrgico de FCVs.

## Abstract (en)

**Objective:**

To identify predictors of kyphoplasty failure in patients with vertebral compression fractures (VCFs), which may assist clinical decision-making and optimize therapeutic outcomes.

**Methods:**

The present retrospective cohort study included patients with VCFs treated by kyphoplasty, categorized based on the presence or absence of treatment failure. Forty records were evaluated with a minimum follow-up time of 6-months, and 6 cases were excluded because they did not meet the inclusion criteria. Variables analyzed included age, sex, time to surgery, fracture level, AO Spine Injury Classification, and osteoporotic fractures (OF) score. Radiological parameters such as vertebral body height, regional and segmental kyphotic angles were measured pre- and postoperatively. Additionally, we assessed the proportion of vertebral body occupied by cement, with emphasis on its anterior and posterior distribution.

**Results:**

Among 34 patients, 20 (58.8%) had successful outcomes, meaning absence of failure criteria until the last follow-up (group 1), while 14 (41.2%) experienced failure (group 2). Failure was defined based on the presence of adjacent vertebral fracture, vertebral re-collapse, or recurrence of incapacitating pain. Kyphoplasty led to significant improvements in vertebral body height and kyphotic angles. In regression analysis, only the posterior cement distribution percentage emerged as an independent predictor of failure (adjusted odds ratio [OR] = 1.684; *p* = 0.012). Increased posterior vertebral height gain showed a trend toward failure, whereas anterior height gain appeared protective.

**Conclusions:**

A higher percentage of cement located posteriorly within the vertebral body is associated with increased kyphoplasty failure risk. These findings highlight the importance of cement distribution patterns in surgical planning for VCFs.

## Introdução

As fraturas por compressão vertebral (FCVs) são relativamente frequentes e, na maioria dos casos, decorrentes de osteoporose. O colapso das vértebras provoca dor aguda e crônica, limita a mobilidade e diminui a qualidade de vida. 1 Estas fraturas podem ser causadas por traumas de baixa energia ou até mesmo por movimentos ou esforços súbitos. De modo geral, as radiografias revelam diminuição da altura do corpo vertebral, redução da radiodensidade, uma cunha anterior e, com menor frequência, acometimento da parede vertebral posterior. A tomografia computadorizada e a ressonância magnética desempenham papéis complementares na avaliação da extensão das fraturas e na identificação de quaisquer complicações neurológicas. 2 O tratamento conservativo é a primeira opção e utiliza analgésicos, órteses ortopédicas, fisioterapia e modificações no estilo de vida. Intervenções mais invasivas podem ser necessárias em caso de dor persistente ou limitações funcionais.

A cifoplastia é uma técnica cirúrgica minimamente invasiva desenvolvida para o tratamento de FCVs. A vertebroplastia é uma alternativa eletiva. A cifoplastia foi introduzida para superar algumas limitações da vertebroplastia, reduzindo a fratura antes da injeção de cimento. A cifoplastia é composta por uma série de etapas e tem como objetivo restaurar a altura vertebral, reduzir os ângulos cifóticos, aliviar a dor e prevenir fraturas subsequentes e outras deformidades da coluna que podem causar complicações em longo prazo, como a cifose. Alguns estudos recentes sugerem que a vertebroplastia e a cifoplastia são equivalentes em proporcionar alívio da dor e melhora da função. 3 A decisão de realizar essas intervenções deve ser ponderada com cuidado, considerando riscos e benefícios. 4 5 Estudos anteriores identificaram o uso de corticosteroides, fraturas na junção toracolombar, baixa densidade mineral óssea (DMO) e extravasamento de cimento como fatores que podem influenciar os desfechos da cifoplastia. 6 7 8 No entanto, muitas variáveis relevantes, como a distribuição do cimento e o aumento diferencial das alturas posterior e anterior, não foram avaliadas, destacando a necessidade de mais estudos sobre os possíveis fatores preditivos de falha da cifoplastia.

Nosso objetivo foi avaliar os resultados da cifoplastia e explorar possíveis fatores preditivos de falha terapêutica. Para tanto, analisamos fatores demográficos e parâmetros relacionados à cirurgia para otimizar o procedimento e seus desfechos.

## Materiais e Métodos

O Comitê de Ética da instituição aprovou e consentiu com este estudo sob número o número 130/2024.

Este é um estudo longitudinal, unicêntrico e analítico de coorte retrospectiva incluindo todos os pacientes submetidos à cifoplastia vertebral percutânea bilateral com balão para tratamento de FCVs em nosso hospital entre 2017 e 2024. Como diversos cirurgiões participaram deste estudo, houve variabilidade técnica, o que pode ter comprometido os resultados. Além disso, não foi possível determinar a quantidade de cimento injetado em cada caso, mas, em média, todos os pacientes receberam 2 a 4 mL do material. Os critérios de inclusão foram histórico de FCV osteoporótica tratada com cifoplastia, idade ≥ 18 anos e período de acompanhamento clínico/radiológico superior a seis meses. Excluímos todos os pacientes com fraturas patológicas e déficits neurológicos.

### Coleta de Dados

Os dados clínicos e demográficos, como idade, sexo, comorbidades associadas, nível da fratura, tipo de fratura (Sistema de Classificação de Lesões da Coluna Vertebral da AO) e classificação de acordo com o Escore de Fraturas Osteoporóticas (OF, do inglês *Osteoporotic Fractures* ), tempo até a cirurgia, tempo de acompanhamento e complicações pós-operatórias, foram coletados dos prontuários dos pacientes. 9 A falha foi definida como dor limitante durante atividades diárias, fratura vertebral adjacente ou novo colapso vertebral.

**Fig. 1:**
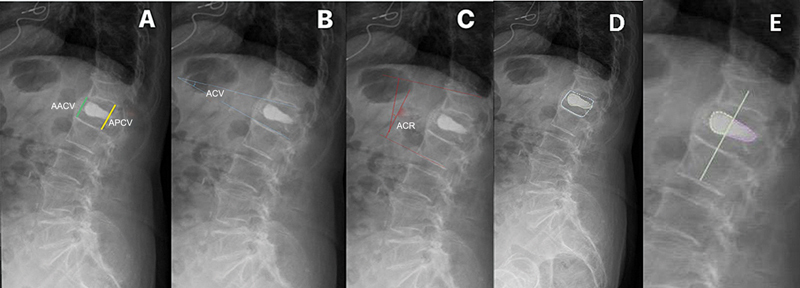
Protocolo de medição da altura anterior do corpo vertebral (AACV), da altura posterior do corpo vertebral (APCV), do ângulo cifótico vertebral (ACV) e do ângulo cifótico regional (ACR). ( **A** ) Protocolo de medição da AACV (linha verde) e da APCV (linha amarela). ( **B** ) Protocolo de medição do ACV (linhas azuis). ( **C** ) Protocolo de medição do ACR usando o ângulo de Cobb (linhas vermelhas). ( **D** ) Medida da área vertebral. ( **E** ) Medida da área de cimento anterior.

Os parâmetros radiológicos medidos antes e depois da cirurgia foram a altura anterior e posterior do corpo vertebral (AACV e APCV, respectivamente), o ângulo cifótico vertebral (ACV) e o ângulo cifótico regional/ângulo de Cobb (ACR) do segmento fraturado 10 ( Fig. 1 ).

Além disso, a distribuição do cimento foi avaliada como a porcentagem da área vertebral ocupada pelo material (porcentagem de área vertebral com cimento) e a porcentagem total do material nas regiões anterior (porcentagem de cimento anterior) e posterior (porcentagem de cimento posterior) da vértebra ( Fig. 1 ). Essas variáveis foram medidas em radiografias em perfil, delimitando a massa de cimento no interior do corpo vertebral utilizando o *software* Sectra (Sectra AB), utilizando valores percentuais para normalizar a medição.

### Análise Estatística

Os dados foram analisados com o programa IBM SPSS Statistics for Windows (IBM Corp.), versão 29.0.

As comparações entre as alturas e os ângulos vertebrais antes e após a cirurgia foram realizadas com o teste t de Student para amostras pareadas, ou com o teste de Wilcoxon em caso de ausência de distribuição normal.

As comparações entre variáveis quantitativas, como idade, alturas e ângulos vertebrais, distribuição do cimento e tempo até a cirurgia, e a falha do procedimento foram realizadas com o teste *t* de Student para amostras independentes, ou o teste de Mann-Whitney para variáveis não paramétricas. A análise da associação entre a falha e as variáveis categóricas, como os escores AO e OF e o nível da fratura, utilizou o teste do qui-quadrado. O teste exato de Fisher foi usado como alternativa ao teste do qui-quadrado em caso de não atendimento às regras de Cochran.

Uma regressão logística multivariada avaliou a contribuição de todas as possíveis variáveis preditoras e a ocorrência de falha da cifoplastia. As variáveis incluídas na regressão foram aquelas com valor de *p* < 0,20. Isso permitiu a criação de um modelo com base nessas variáveis para explicação parcial dos desfechos.

O nível de significância foi estabelecido como *p* < 0,05 em todos os testes.

## Resultados

Trinta e quatro pacientes atenderam aos critérios e foram incluídos neste estudo. Destes, 20 casos (58,8%) foram incluídos no grupo 1, definido pela ausência de falha. Catorze casos (41,2%) foram incluídos no grupo 2 por apresentarem complicações ( Tabela 1 ).

**Tabela 1:** Características dos grupos 1 e 2

	Grupo 1	Grupo 2
**Número de pacientes**	20	14
**Idade (anos)**	70.95 ± 13.59	72.71 ± 9.05
**Sexo**		
**Masculino**	5 (25%)	5 (35,71%)
**Feminino**	15 (75%)	9 (64,29%)
**Segmento fraturado**		
**D6**	1 (5%)	0
**D9**	1 (5%)	0
**D10**	1 (5%)	2 (14,3%)
**D11**	3 (15%)	1 (7,1%)
**D12**	4 (20%)	3 (21,4%)
**L1**	3 (15%)	4 (28,6%)
**L2**	2 (10%)	0
**L3**	5 (25%)	3 (21,4%)
**L4**	0	1 (7,1%)
**Tempo entre a lesão e a cirurgia (meses)**	6 (5)	6 (6)
**AACV pré-operatória (cm)**	1,72 ± 0,49	1,87 ± 0,62
**APCV pré-operatória (cm)**	2,64 ± 0,51	2,61 ± 0,58
**ACV pré-operatório (graus)**	13,71 ± 7,3	11,36 ± 6,36
**ACR pré-operatório (graus)**	18,23 ± 6,41	19,21 ± 6,42
**AACV pós-operatória (cm)**	2,07 ± 0,39	2,07 ± 0,6
**APCV pós-operatória (cm)**	2,84 ± 0,464	2,95 ± 0,39
**ACV pós-operatório (graus)**	10,04 ± 6,7	11,29 ± 6,39
**ACR pós-operatório (graus)**	16,39 ± 15,16	18,47 ± 9,11
Variação percentual da AACV ([pós–pré-operatória]*100)/pré-operatória)	20,85 (22,29)	12,32 (12,35)
Variação percentual da APCV ([pós–pré-operatória]*100)/pré-operatória)	2,36 (18,61)	9,843 (23,29)
**Diferença do ACR (pós–pré-operatório) (graus)**	1,75 (4,15)	1,55 (2,37)
**Área vertebral com cimento (%)**	44,34 ± 10,20	44,93 ± 10,43
**Cimento anterior (%)**	69,25 (10,52)	48,80 (9,61)
**Cimento posterior (%)**	38,14 ± 6,85	51,57 ± 6,66
**AO**		
**A1**	18 (90%)	13 (91,2%)
**A2**	1(5%)	1 (2,9%)
**A3**	1(5%)	2 (5,9%
**Escore OF**		
**OF1**	1 (5%)	0
**OF2**	16 (80%)	10 (71,4%)
**OF3**	2 (10%)	5 (14,7%)
**OF4**	1 (5%)	2 (5,9%)

**Abreviaturas:** AACV, altura anterior do corpo vertebral; APCV, altura posterior do corpo vertebral; ACR, ângulo cifótico regional; ACV, ângulo cifótico vertebral; AO, Sistema de Classificação de Lesões da Coluna Vertebral da AO; OF, fraturas osteoporóticas.

**Nota:** Resultados expressos como n (%), média ± desvio padrão ou mediana (intervalo interquartil [IIQ]).

A dor incapacitante foi a causa mais frequente de falha do procedimento (35,3%). A Tabela 2 mostra a prevalência de cada causa.

**Tabela 2:** Frequências dos tipos de falha após o procedimento

Tipo de falha após o procedimento	
Nenhum	20 (58,8%)
Dor incapacitante	12 (35,3%)
Novo colapso vertebral	1 (2,9%)
Fratura de vértebra adjacente	1 (2,9%)
Extravasamento de cimento	0

Ao comparar os valores gerais antes e depois do procedimento, a APCV ( *p* < 0,001), o ACV ( *p* < 0,001), a AACV ( *p* < 0,001) e o ACR ( *p* = 0,003) apresentaram diferenças significativas entre as duas avaliações, com uma tendência de aumento da APCV e da AACV e diminuição do ACV e do ACR após a cirurgia. Estes achados corroboram a validade da cifoplastia como método para restaurar a altura e a morfologia normais do corpo vertebral. Outras análises estatísticas são mostradas na Tabela 3 .

**Tabela 3:** Avaliação das variáveis antes e após a intervenção cirúrgica

	Antes da cirurgia	Após a cirurgia	*p*	Tamanho do efeito
APCV M (DP)	2,63 (0,53)	2,89 (0,43)	**< 0,001**	*d =* 0,54
ACV M (DP)	12,74 (6,93)	10,55 (6,5)	**< 0,001**	*d =* −0,44
AACV Mdn (IIQ)	1,74 (0,64)	2,02 (0,67)	**< 0,001**	*r =* 0,56
ACR Mdn (IIQ)	19,06 (13,2)	17,25 (14,3)	**0,003**	*r =* −0,36

**Abreviaturas** : AACV, altura anterior do corpo vertebral; ACR, ângulo cifótico regional; ACV, ângulo cifótico vertebral; APCV, altura posterior do corpo vertebral; DP, desvio padrão; IIQ, intervalo interquartil; M, média; Mdn, mediana.

**Nota:** Resultados expressos como n (%), média ± desvio padrão ou mediana (IIQ).

Não houve relação estatisticamente significativa entre sexo, escore OF, classificação AO e nível da fratura e a falha do procedimento. No entanto, no grupo com falha (grupo 2), observou-se uma porcentagem maior de pacientes do sexo masculino (35,7%) em comparação ao grupo 1 (25,0%), embora sem significância estatística ( *p* = 0,704). Em relação ao escore OF, a classificação mais frequente em ambos os grupos foi OF 2 (80% no grupo 1 e 71,4% no grupo 2).

As fraturas de tipo A1 foram as mais frequentes em ambos os grupos; as fraturas lombares foram mais prevalentes no grupo com falha (57,1%), porém sem diferenças estatisticamente significativas. Estes resultados são apresentados na Tabela 4 .

**Tabela 4:** Associação entre as variáveis categóricas deste estudo e a falha do procedimento

	Sem falha	Com falha	*p*	Tamanho do efeito
**Sexo***n* (%)			0,704	Φ = −0,12
Masculino	5 (25,0)	5 (35,7)		
Feminino	15 (75,0)	9 (64,3)		
**Escore OF***n* (%)			0,889	Φ _c_ = 0,215
OFS1	1 (5,0)	0		
OFS 2	16 (80,0)	10 (71,4)		
OFS 3	2 (10)	3 (21,4)		
OFS 4	1 (5,0)	1 (5,0)		
**AO***n* (%)			1	Φ _c_ = 0,151
A1	18 (90,0)	13 (92,9)		
A2	1 (5,0)	0		
A3	1 (5,0)	1 (7,1)		
**Nível da fratura***n* (%)			0,738	Φ = −0,70
Torácico	10 (50,0)	6 (42,9)		
Lombar	10 (50,0)	8 (57,1)		

**Abreviaturas:** n, frequência absoluta; Φ, tamanho do efeito; Φ _c_ , V de Cramer; AO, Sistema de Classificação de Lesões da Coluna Vertebral da AO; OF, fraturasosteoporóticas.

**Nota:** Resultados expressos como n (%), média ± desvio padrão ou mediana (intervalo interquartil [IIQ]).

Ao comparar as variáveis radiológicas com a falha, o grupo 2 apresentou porcentagem maior de distribuição de cimento nas regiões posteriores da vértebra (51,7% *versus* 38,1%; *p* < 0,001) e porcentagem menor de cimento na metade anterior da vértebra (48,8% *versus* 69,3%; *p* < 0,001), ambas estatisticamente significativas. Embora as demais variáveis não tenham apresentado diferenças estatisticamente significativas, a variação percentual da APCV teve significância marginal ( *p* = 0,163).

Os resultados são mostrados na Tabela 5 .

**Tabela 5:** Associação entre as variáveis estudadas e a falha do tratamento

	Sem falha (n = 20)	Com falha (n = 14)	*p*	Tamanho do efeito
Idade M (DP)	70,95 (13,59)	72,71(9,05)	0,675	*d = 0,15*
Tempo entre a lesão e a cirurgia (meses) Mdn (IIQ)	6 (5)	6 (6)	0,958	*r* = 0,16
AACV pré-operatória (cm) M (DP)	1,72 ± 0,11	1,87 ± 0,17	0,449	*d = 1,05*
APCV pré-operatória (cm) M (DP)	2,64 ± 0,11	2,60 ± 0,16	0,861	*d = 0,36*
ACV pré-operatório (graus) M (DP)	13,71 ± 1,63	11,36 ± 1.70	0,340	*d = 1,41*
ACR pré-operatório (graus) M (DP)	18,23 ± 3,78	19,21 ± 1,72	0,813	*d = 0,33*
AACV pós-operatória (cm) M (DP)	2,08 ± 0,09	2,07 ± 0,16	0,984	*d = 0,07*
APCV pós-operatória (cm) M (DP)	2,84 ± 0,10	2,95 ± 0,11	0,462	*d = 1,05*
ACV pós-operatório (graus) M (DP)	10,04 ± 1,49	11,29 ± 1,71	0,589	*d = 0,78*
ACR pós-operatório (graus) M (DP)	16,39 ± 3,39	18,47 ± 2,44	0,650	*d = 0,7*
Variação da AACV (%) Mdn (IIQ)	20,85 (22,29)	12,32 (12.35)	0,221	*r* = 0,21
Variação da APCV (%) M (DP)	2,36 (18,61)	9,84 (23,29)	0,163	*d = 0,35*
Diferença do ACR (graus) Mdn (IIQ)	1,75 (4,15)	1,55 (2,37)	0,587	*r* = 0,09
Área vertebral com cimento (%) M (DP)	44,34 ± 10,20	44,93 ± 10,43	0,869	*d =* 0,06
Cimento anterior (%) Mdn (IIQ)	69,25 (10,52)	48,80 (9,61)	**0,001**	*r* = - 0,80
Cimento posterior (%) M (DP)	38,14 ± 6,85	51,57 ± 6,66	**0,001**	*d* = 6,77

**Abreviaturas:** AACV, altura anterior do corpo vertebral; ACR, ângulo cifótico regional; ACV, ângulo cifótico vertebral; APCV, altura posterior do corpo vertebral; DP, desvio padrão; IIQ, intervalo interquartil; M, média; Mdn, mediana.

**Nota:** Resultados expressos como n (%), média ± desvio padrão ou mediana (IIQ).

### Modelos de Regressão Logística

A seguir, os modelos de regressão logística foram desenvolvidos. As variáveis que apresentaram diferenças estatisticamente significativas em relação à falha da cifoplastia e valores de *p* < 0,20 foram incluídas. Assim, a porcentagem de cimento anterior, a porcentagem de cimento posterior e a variação percentual da APCV foram adicionadas aos modelos. Uma vez que a porcentagem de cimento anterior e a porcentagem de cimento posterior apresentaram colinearidade significativa, dois modelos distintos de regressão foram desenvolvidos com essas variáveis.

O primeiro modelo incluiu as seguintes variáveis: variação percentual da APCV e a porcentagem de cimento posterior. Primeiro, realizamos um teste Omnibus, que revelou que o modelo era estatisticamente significativo ( *p* < 0,001). Em seguida, avaliamos o R ^2^ de Nagelkerke, que indicou que as duas variáveis na regressão explicavam 75,1% dos desfechos. Das duas variáveis analisadas, apenas a porcentagem de cimento posterior mostrou ser um fator preditivo independente e estatisticamente significativo de falha do tratamento (razão de chances [RC] ajustada = 1,775; *p* = 0,022). Ao utilizar apenas a porcentagem de cimento posterior, o modelo também foi estatisticamente significativo ( *p* < 0,001), e o R ^2^ de Nagelkerke indicou que a variável na regressão explicava 74,5% dos resultados. A porcentagem de cimento posterior apresentou associação estatisticamente significativa com a falha cirúrgica (OR ajustada = 1,684; *p* = 0,012).

Uma regressão logística também foi realizada usando a porcentagem de cimento anterior, mas o modelo mostrou uma associação estatisticamente não significativa com a falha.

## Discussão

As FCVs são uma causa relativamente frequente de dor aguda e crônica e limitações funcionais, em especial em pacientes idosos, com impacto significativo na qualidade de vida. O tratamento pode incluir medidas conservadoras e, em alguns casos, procedimentos cirúrgicos, como técnicas percutâneas, tais como cifoplastia e vertebroplastia. De modo geral, o tratamento cirúrgico é escolhido após o insucesso das medidas conservadoras. No entanto, a decisão de submeter um paciente à cirurgia deve ser ponderada com cuidado. Portanto, compreender os fatores de risco e identificar possíveis fatores preditivos de falha cirúrgica é essencial para orientar a tomada de decisões clínicas e melhorar os desfechos do paciente. 11

Tem sido defendido que a cifoplastia gera maior grau de restauração da altura ou redução do ângulo cifótico; porém, muitos pesquisadores consideram que os primeiros resultados clínicos da cifoplastia e da vertebroplastia são semelhantes. 12 Du et al., 3 por exemplo, em uma coorte de 112 pacientes com FCVs dolorosas, compararam os resultados clínicos e radiológicos da cifoplastia e da vertebroplastia e concluíram que os desfechos clínicos das duas intervenções são semelhantes nos dois primeiros anos após a cirurgia. Em outros dois trabalhos, publicados por Liu et al. 13 e Cheng et al., 14 os desfechos clínicos entre os dois grupos foram similares, levando os autores a recomendarem a vertebroplastia em vez da cifoplastia devido ao maior custo do procedimento com balão cifótico.

Apesar dos desfechos clínicos equivalentes da cifoplastia e da vertebroplastia, diversos artigos demonstraram que a cifoplastia bipedicular (CBP) proporciona melhor restauração da altura vertebral, maior redução do ângulo cifótico e menor extravasamento de cimento. 3 15 16 Gan et al., 17 em um estudo com 38 pacientes, e Kim et al., 18 com um total de 103 pacientes com FCV osteoporótica, demonstraram que a CBP apresentou vantagem significativa sobre a vertebroplastia em termos de restauração da altura vertebral média. Röllinghoff et al., 19 em um estudo prospectivo com 90 pacientes com FCVs osteoporóticas recentes, concluíram que a restauração média da altura do corpo vertebral após um ano de acompanhamento foi significativamente maior ( *p* < 0,05) no grupo submetido à cifoplastia. Alguns artigos também demonstraram que a vertebroplastia esteve associada a uma maior taxa de complicações. 19 20

Este estudo avaliou os resultados da cifoplastia percutânea em uma coorte de 34 pacientes com FCVs. Nossos resultados indicaram que 58,8% dos pacientes não apresentaram falhas durante o acompanhamento, enquanto 41,2% apresentaram falha cirúrgica em diferentes momentos. A taxa de falha observada é notável; a recorrência da dor foi o tipo de falha mais frequente, ocorrendo em 35,3% dos casos. Hackbarth et al. 21 observaram taxa semelhante de recorrência da dor em longo prazo (30,9%) em seu estudo com 49 pacientes submetidos à cifoplastia pelo menos 3 meses após a intervenção. Esses resultados enfatizam a complexidade do tratamento das FCVs e a variabilidade dos desfechos.

A literatura indica diversas complicações associadas à cifoplastia, como radiculopatias, fraturas de costelas, extravasamento de cimento e compressão da medula espinhal ou de nervos. Em nosso estudo, porém, não observamos nenhuma dessas complicações. Além disso, não detectamos infecções relacionadas ao procedimento, problemas cardíacos ou pneumotórax em nossos pacientes. 22

Em nosso estudo, os parâmetros radiológicos AACV, APCV, ACV e ACR melhoraram de forma significativa, com aumento da altura vertebral e diminuição dos ângulos cifóticos vertebrais. Diversos estudos corroboraram a eficácia da cifoplastia. De Falco et al., 10 utilizando 3 medidas de altura do corpo vertebral (anterior, média e posterior), em seu estudo com 61 pacientes submetidos à cifoplastia, demonstraram boa recuperação destes 3 parâmetros, que aumentaram de maneira estatisticamente significativa após o tratamento. O estudo também mostrou uma variação maior na AACV do que na APCV, o que está de acordo com nossos achados. 10 Lieberman et al., 23 em um estudo com 30 pacientes, constataram que o procedimento restaurou 47% da altura perdida em 70% dos corpos vertebrais. Assim, houve recuperação substancial da altura após a cifoplastia, comprovando sua eficácia no tratamento de FCVs.

Em relação à recuperação da altura vertebral, nossa pesquisa revelou alguns resultados inesperados que merecem maior discussão e devem ser considerados, uma vez que podem influenciar significativamente os desfechos dos pacientes. Ao examinarmos as variáveis entre os 2 grupos, constatamos que a variação percentual da AACV foi de 20,85% no grupo 1 (sem falha) e de 12,32% no grupo 2 (com falha). Esses achados corroboram a expectativa de que maiores melhoras na altura do corpo vertebral estejam correlacionadas a desfechos superiores apesar da ausência de significância estatística. Em contrapartida, as variações percentuais da APCV apresentaram diferença de 9,84% no grupo com falha, em comparação a apenas 2,36% no grupo sem falha, questionando a suposição de que o aumento da altura do corpo vertebral melhoraria os desfechos. Isso levanta a hipótese de que um aumento nas variações da APCV pode ser associado a piores resultados em pacientes submetidos à cifoplastia. Embora não tenha havido significância estatística, acreditamos que o pequeno tamanho da amostra represente uma limitação importante e que uma investigação mais aprofundada, com amostras maiores, pode vir a confirmar a tendência suspeita. Até onde temos conhecimento, não existem referências a essas hipóteses na literatura.

Nosso estudo não encontrou associação significativa entre a falha da cifoplastia e diversas variáveis demográficas e clínicas, incluindo sexo, idade, tempo decorrido entre a lesão e a cirurgia e nível da fratura. Este achado contrasta com pesquisas anteriores que sugeriram que a idade avançada poderia influenciar os desfechos cirúrgicos. Morozumi et al. 24 mostraram que pacientes idosos apresentavam maior risco de desenvolvimento de fraturas vertebrais adjacentes, com médias de idade de 80 e 76 anos no grupo acometido e não acometido, respectivamente. Contudo, nossos resultados corroboram a hipótese de que os aspectos técnicos dos procedimentos podem ser mais importantes do que essas variáveis demográficas na determinação do sucesso cirúrgico.

Observamos uma associação estatisticamente significativa entre a porcentagem de cimento na metade anterior ou posterior das vértebras e as falhas do tratamento. Especificamente, uma maior porcentagem de cimento na região posterior foi um fator preditivo independente de falha. Isso sugere que o aumento da quantidade de cimento na região posterior está associado a um maior risco de falha do tratamento. Com base nesses resultados, levantamos a hipótese de que a colocação inadequada do cimento, em especial na região posterior, compromete a integridade estrutural da vértebra, aumentando o risco de complicações futuras. Esses achados talvez corroborem a hipótese anterior de que uma maior variação na APCV pode provocar falha do procedimento, possivelmente pelo aumento da cifose local.

Há diversos estudos sobre a distribuição do cimento na cifoplastia, mas, até onde sabemos, poucos a avaliaram nas regiões anterior e posterior. He et al. 25 relataram que a ampla distribuição de cimento pode melhorar o ângulo cifótico e a altura vertebral, sem causar complicações. Em nosso estudo, porém, a posição do cimento no interior do corpo vertebral não foi discriminada, impossibilitando sua correlação com nossos achados.

Tan et al., 26 em um estudo com 137 pacientes com fratura vertebral osteoporótica de um único nível e submetidos à cirurgia percutânea, divididos com base na distribuição do cimento em grupo A (com contato completo do cimento com ambas as placas terminais da vértebra) e grupo B (com contato parcial), mostraram que o grupo A apresentou resultados melhores em termos de alívio da dor, restauração da altura vertebral e menor taxa de recompressão. Considerando esses achados e combinando-os com as evidências encontradas em nosso estudo, a importância da distribuição ideal do cimento durante a cifoplastia não pode ser subestimada, visto que comprovadamente tem impacto direto nos desfechos do paciente. 26

Embora este estudo ofereça informações valiosas, algumas limitações devem ser reconhecidas. Primeiramente, a pesquisa foi conduzida em uma única instituição médica e baseou-se em dados retrospectivos, o que pode gerar preocupações quanto à precisão e generalização dos dados. Em segundo lugar, existem algumas dificuldades em medir um objeto tridimensional, como o corpo vertebral, a partir de imagens bidimensionais. A altura do corpo vertebral pode variar entre a face lateral e a face medial, o que pode comprometer a precisão das medidas. Além disso, o tamanho limitado da amostra restringiu o poder estatístico e a generalização dos resultados, podendo afetar sua robustez e confiabilidade, tornando crucial sua interpretação cautelosa. Pesquisas futuras com coortes maiores são essenciais para validar esses achados e explorar outros fatores que podem influenciar os desfechos cirúrgicos. Por fim, há uma escassez de estudos similares na literatura, o que dificulta a obtenção de conclusões definitivas ou a realização de comparações mais significativas.

## Conclusão

Em resumo, nossos achados corroboram a evidência de que a cifoplastia é uma opção viável e eficaz para o tratamento de FCVs e a restauração da morfologia vertebral normal. Além disso, este estudo identificou a porcentagem de cimento distribuída na região posterior do corpo vertebral como um fator preditivo significativo de falhas cirúrgicas, sugerindo que pode contribuir para o insucesso da cifoplastia em pacientes com FCVs.

A identificação de fatores específicos associados à falha cirúrgica, particularmente em relação à distribuição do cimento, enfatiza a necessidade de técnicas cirúrgicas meticulosas para otimização dos desfechos. Portanto, sugerimos que a distribuição cuidadosa do cimento durante a cifoplastia, com sua aplicação predominante na região anterior das vértebras, pode levar a melhores resultados e prevenir a falha cirúrgica.

Pesquisas contínuas nesta área, com número maior de participantes, são essenciais para refinar os critérios de seleção de pacientes e explorar os resultados sugeridos em profundidade, a fim de aumentar as taxas de sucesso em longo prazo da cifoplastia e, em última análise, melhorar os desfechos dos pacientes e sua qualidade de vida.
